# Antioxidative Activity of Remimazolam as a Direct Free Radical Scavenger in Comparison With Midazolam

**DOI:** 10.7759/cureus.94676

**Published:** 2025-10-15

**Authors:** Shigekiyo Matsumoto, Yuta Ukon, Shiena Endo, Mika Sasaki, Yoshifumi Ohchi, Yoshimasa Oyama, Tetsuya Uchino, Chihiro Shingu, Kazue Ogata, Osamu Tokumaru

**Affiliations:** 1 Anesthesiology, Oita University, Oita, JPN; 2 Medicine, Oita University, Oita, JPN; 3 Welfare and Health Sciences, Oita University, Oita, JPN

**Keywords:** antioxidative effects, electron spin resonance, free radical, midazolam, remimazolam

## Abstract

Introduction

Remimazolam, a novel ultra-short-acting benzodiazepine, has seen increasing use in recent years as a general anesthetic for patients with poor cardiac function and older adults due to its hemodynamic stability and rapid recovery profile. Alternatively, it has also become clear through several basic and clinical studies that it suppresses inflammation and oxidative stress. However, its direct free-radical-scavenging activity remains largely unreported. In this study, we aimed to evaluate its direct non-enzymatic scavenging activity against multiple free radical species.

Materials and methods

A constant amount of eight types of free radicals or singlet oxygen, a reactive oxygen species, was generated in disposable cells. After adding various concentrations of remimazolam to the free radical generation system, the radical scavenging activity of the drug was measured using electron spin resonance spectroscopy with the spin-trapping method. Concentration-response curves for the scavenging activity against free radicals and singlet oxygen were constructed. The 50% inhibitory concentration (IC_50_) values derived from these curves, and the concentrations of spin traps, were used to estimate the reaction rate constants between remimazolam and free radicals as indicators of the scavenging activity of remimazolam. The IC_50_ of midazolam against free radicals was also evaluated for comparison.

Results

Remimazolam scavenged hydroxyl radicals, superoxide anions, ascorbyl free radicals, and singlet oxygen, with reaction rate constants of 4.2×10^10^ (standard error=1.5×10^10^) M^-1^s^-1^, 8.6 (5.5) *k_G_*_-CYPMPO_, 0.31 (0.13) *k*_edaravone,_ and 1.9×10 (3) *k*_4-OH_TEMP_, respectively. However, remimazolam did not scavenge nitric oxide, 2,2-diphenyl-1-picrylhydrazyl, or tyrosyl radicals. The reaction rate constant of remimazolam toward the hydroxyl radical was comparable to that of the radical scavenger edaravone (5.2×10¹⁰ M⁻¹s⁻¹). Remimazolam had a higher reaction rate constant against hydroxyl radical than midazolam (1.3×10^8^ M⁻¹s⁻¹).

Discussions

Remimazolam exhibited a higher radical-scavenging activity than midazolam against multiple free radicals. Notably, remimazolam possesses high scavenging activity, comparable to that of edaravone, particularly against the highly injurious hydroxyl radical. It also demonstrated scavenging activity against the superoxide anion, a reactive oxygen species produced upstream of the chain reactions of free radical generation during ischemia-reperfusion injury. These properties of remimazolam potentially offer beneficial antioxidant effects in anesthetic management.

## Introduction

Remimazolam is a benzodiazepine-derivative intravenous anesthetic approved for clinical use in Japan in 2020 for the induction and maintenance of general anesthesia [1]. It has superior clinical properties compared to the current mainstream propofol, such as less circulatory depression, lower risk of bacterial growth due to the absence of fat droplets in the solvent, and reduced likelihood of causing injection pain [2].

Additionally, remimazolam reduces inflammation and oxidative stress. In animal studies, the antioxidative effects of remimazolam were demonstrated in ischemia-reperfusion models of the liver, heart, and brain [3-5]. In clinical studies, remimazolam increased the levels of antioxidant enzymes, such as superoxide dismutase (SOD) and glutathione peroxidase (GSH-Px), in the blood of patients undergoing isolated lung ventilation, thereby reducing oxidative stress [6]. However, its direct free radical scavenging activity remains largely unreported [7].

Recently, accumulating evidence from measurements of perioperative oxidative stress markers and other methods has revealed that oxidative stress contributes to the pathogenesis of postoperative delirium (POD) in addition to previously reported neuroinflammation [8, 9]. Risk factors for POD include advanced age, preoperative cognitive impairment, severity of the primary disease requiring surgery, preoperative comorbidities, and polypharmacy [10, 11]. Furthermore, long-term administration of benzodiazepines increases the risk of developing POD, even under normal anesthetic conditions [10]. Although a few reports have indicated an association between intraoperative benzodiazepine use and the onset of POD, the perioperative use of benzodiazepine drugs requires caution in patients with multiple POD risk factors [12]. Interestingly, meta-analyses reported that remimazolam, a benzodiazepine derivative, has an incidence rate of POD similar to that of propofol [13, 14].

We speculated that remimazolam might reduce the incidence of POD through its antioxidant effects equivalent to those of propofol and might suppress oxidative stress more strongly than any other benzodiazepine clinically available at present, although the mechanism is unclear.

In this study, we hypothesized that remimazolam directly scavenges specific free radicals in a dose-dependent manner, contributing to its antioxidant activity. We evaluated its direct non-enzymatic scavenging activity against multiple free radical species.

## Materials and methods

The scavenging activity of remimazolam was evaluated against the following eight species of free radicals and singlet oxygen by electron spin resonance (ESR) spectroscopy using the spin-trapping method: oxygen-centered radicals (hydroxyl radical, superoxide anion, tert-butyl peroxyl radical, tert-butoxyl radical, and ascorbyl free radical), nitrogen-centered radicals (nitric oxide and 2,2-diphenyl-1-picrylhydrazyl (DPPH)), amino acid radicals (tyrosyl radical), and reactive oxygen species (singlet oxygen).

Materials

The active pharmaceutical ingredient, remimazolam, was generously provided by Mundipharma International Ltd. (Cambridge, UK). Tert-butyl hydroperoxide and DPPH were purchased from Sigma-Aldrich (St. Louis, MO, USA). Reagents such as 5,5-dimethyl-1-pyrroline-N-oxide (DMPO), 1-hydroxy-2-oxo-3-(N-methyl-3-aminopropyl)-3-methyl-1-triazene (NOC7), and 2-(4-carboxyphenyl)-4,4,5,5-tetramethylimidazoline-1-oxyl 3-oxide (carboxy-PTIO) were purchased from Dojindo (Kumamoto, Japan). Midazolam, sodium ascorbate, dimethyl sulfoxide, and hydrogen peroxide were purchased from FUJIFILM Wako Pure Chemical Corporation (Osaka, Japan). Chemicals such as 2,2′-azobis (2-amidinopropane) dihydrochloride (AAPH), acid red 94, and 4-hydroxy-2,2,6,6-tetramethylpiperidine (4-OH TEMP) were purchased from Tokyo Chemical Industry (Tokyo, Japan). The gauche form 5-(2,2-dimethyl-1,3-propoxy cyclophosphoryl)-5-methyl-1-pyrroline N-oxide (G-CYPMPO) was generously provided by Dr. M. Kamibayashi [15]. All other reagents were of the highest commercially available quality.

ESR spectrometry

ESR spectrometry was conducted as previously described [16-19]. Free radicals were quantified using an X-band ESR spectrometer (JES-RE1X; JEOL, Tokyo, Japan) and a WIN-RAD ver. 1.20b (Radical Research Inc., Tokyo, Japan). The ultraviolet (UV)/visible (VIS) light source was a 200 W medium-pressure mercury-xenon arc lamp (UVF-203S, San-Ei Electric, Osaka, Japan) with either UV-transmitting/VIS-absorbing filter or vice versa. Free radicals were produced in disposable glass micro-hematocrit capillary tubes (CS-HMT-502; Chase Scientific, Fort Lauderdale, USA). The typical instrument settings were as follows: room temperature (23 °C); frequency 9.45 GHz with 100-kHz modulation; modulation width, 0.1 mT; time constant, 0.1 s; center field, 335.8 mT; sweep width, 7.5 mT; sweep time, one minute. The microwave power was set at 4 mW to avoid saturation of the ESR signals.

Table 1 lists the production and trapping methods of free radicals. Briefly, hydroxyl radicals were produced using a Fenton-type reaction and trapped by G-CYPMPO. Superoxide anions were generated by mixing hypoxanthine and xanthine oxidase, and were trapped by G-CYPMPO. Tert-butyl peroxyl radicals were produced by UV irradiation of tert-butyl hydroperoxide and trapped by G-CYPMPO. Tert-butoxyl radical was generated by UV irradiation with 1 mM AAPH and trapped by G-CYPMPO. Ascorbyl free radicals were produced by adding 99% dimethyl sulfoxide to sodium ascorbate [20]. Singlet oxygen was generated by VIS light irradiation of Acid Red 94 with its quencher 4-OH TEMP, forming a 4-hydroxy-2,2,6,6-tetramethylpiperidine-1-oxyl radical. The nitric oxide radicals produced from NOC7 reacted with carboxy-PTIO to generate carboxy-PTI, which was measured 60 min after mixing. DPPH, a stable artificial free radical, was directly quantified by ESR. Tyrosyl radicals were generated from hemoglobin by adding hydrogen peroxide and trapped by DMPO.

**Table 1:** Production and trapping systems of free radicals AAPH - 2-2'-Azobis (2-amidinopropane) dihydrochloride; carboxy-PTIO - 2-(4-carboxypheyl)-4,4,5,5-tetramethylomidazoline-1-oxyl 3-oxide; G-CYPMPO - Gauche form 5-(2,2-dimethyl-1,3-propoxy cyclophosphoryl)-5-methyl-1-pyrroline N-oxide; DMPO - 5,5-dimethyl-1-pyrroline-N-oxide; DMSO - dimethyl sulfoxide; DPPH - 2,2-diphenyl-1-picrylhydrazyl; DTPA - diethylenetriaminepentaaceticacid; NOC7 - N-methyl-3-(1-methyl-2-hydroxy-2-nitrosohydrazino)-1-propanamine; 4-OH TEMP - 4-hydroxy-2,2,6,6-tetramethylpiperidine

Free radical species	Precursor/sensitizer	Spin trap/quencher
Hydroxyl radical	0.015% hydrogen peroxide + 50 μM FeSO_4_	2.5 mM G-CYPMPO
Superoxide anion	0.04 mM hypoxanthine + 0.02 U/mL xanthine oxidase	5 mM G-CYPMPO
tert-Butyl peroxyl radical	80 mM tert-butyl hydroperoxide + 0.1 mM DTPA + UV 15 s	5 mM G-CYPMPO
tert-Butoxyl radical	1.4 mM AAPH + UV 4 s	3.8 mM G-CYPMPO
Ascorbyl free radical	0.26 mg/ml sodium ascorbate + 42% DMSO	―
Singlet oxygen	0.17 mM Acid red + 500-600 nm light 60 s	1.7 mM 4-OH TEMP
Nitric oxide	140 μM NOC7	14 μM carboxy-PTIO
DPPH	15 μM DPPH	―
Tyrosyl radical	0.11 mM myoglobin + 0.002% hydrogen peroxide	600 mM DMPO

The third and fourth ESR signals of manganese (II) oxide (MnO) were used as external references for ascorbyl free radical, nitric oxide, DPPH, and tyrosyl radical. Since the ESR spectra of the spin adducts of G-CYPMPO overlapped with those of the third and fourth peaks of the MnO spectrum, the second and fifth peaks of MnO were used for the G-CYPMPO-spin adducts when detecting hydroxyl radicals, superoxide anions, tert-butyl peroxyl radicals, and tert-butoxyl radicals.

It is customary to present ESR spectra as differential spectra, as in this study. Given that the line width remains unchanged, the ratios of the signal heights in the differential ESR spectrum correspond to the ESR signal intensities [18] and thus to the amount or concentration of free radicals. The ratio of the target free radical signal to that of MnO was calculated and standardized relative to the control ESR signal without remimazolam. 

Calculation of 50% inhibitory concentration (IC_50_)

The concentration-response curves were calculated non-parametrically by fitting the data to the Cheng-Prusoff equation [21]:

 $y=\frac{1}{1+(\frac{x}{a})^b}$

where a is the estimated IC_50_, *x* is the final concentration of remimazolam, and *y* is the observed free radical activity relative to the control. 

Estimation of reaction rate constants 

According to the kinetic competition model, the following competitive reactions occur in the reaction mixture, with the reaction rate constants provided in parentheses:

spin trap + free radical --> spin-adduct (*k*_trap_)

remimazolam + free radical --> remimazolam -radical (*k*_RMZ_)

where *k*_trap_ and *k*_RMZ_ are the second-order rate constants of the spin trap and remimazolam, respectively. Because the two reactions above are in equilibrium at IC_50_, the reaction rate constant *k*_RMZ_ is given as follows:

${\it k}_{\rm RMZ}=\frac{\rm [spin trap]}{\rm IC_{50}}k_{\rm trap}$

*k*_trap_ used were as follows: *k*_G-CYPMPO_ for hydroxyl radical 4.2 × 10^9^ M^-1^s^-1^ [15], and *k*_c-PTIO_ for nitric oxide 1.01 × 10^4^ M^-1^s^-1^ [22]. Because *k*_G-CYPMPO_ for superoxide anions, tert-butyl hydroperoxide, and tert-butoxyl radicals have not been reported, *k*_RMZ_ for these free radicals is presented relative to *k*_G-CYPMPO_. The reaction rate constants of midazolam (*k*_MDZ_) were also estimated based on IC_50_.

Statistical analysis 

Statistical analyses were performed using R ver. 4.3.0 (R Foundation, Vienna, Austria). Values are presented as estimated means and 95% confidence intervals. The level of significance was set at 0.05.

## Results

**Figure 1:**
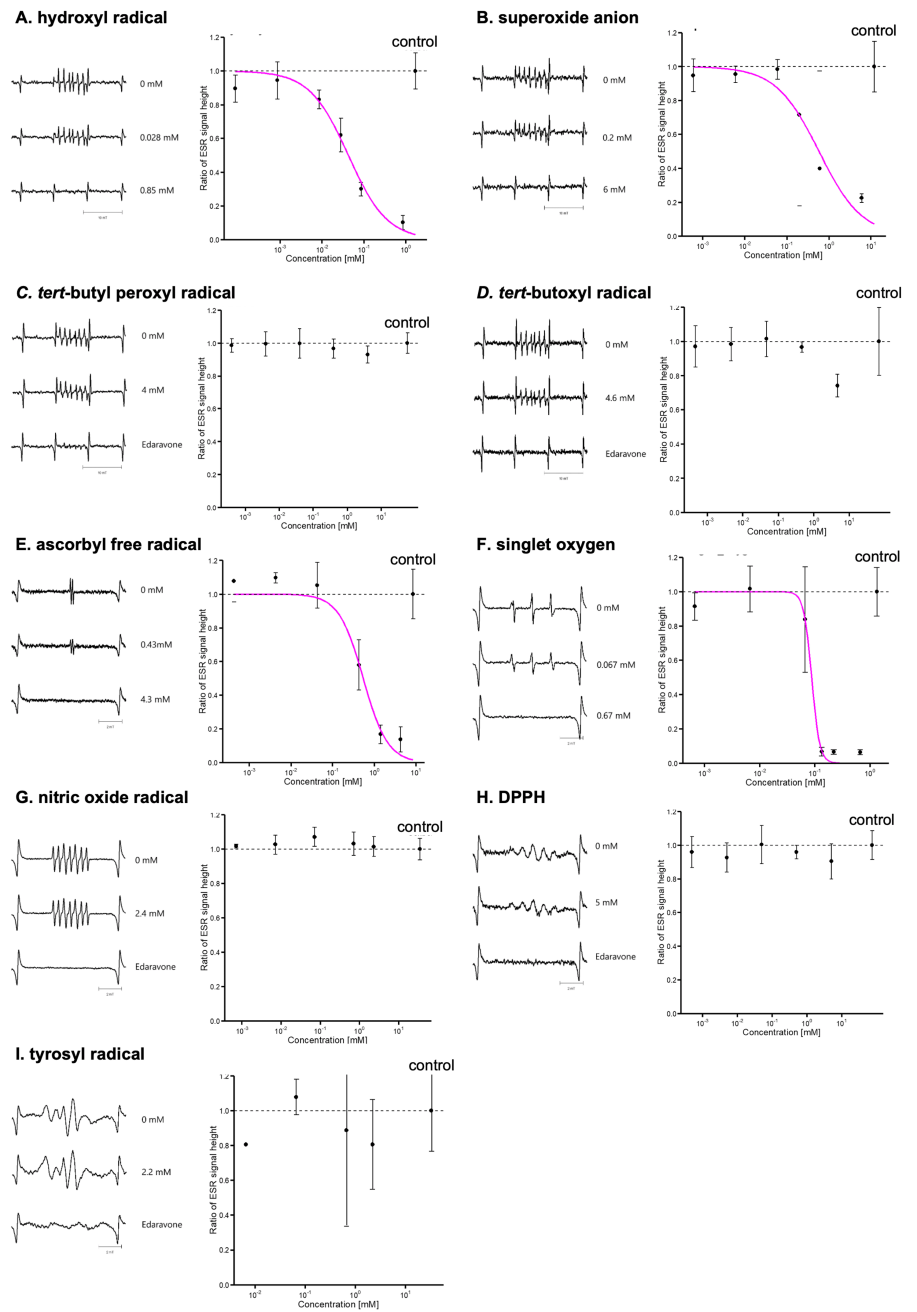
Electron spin resonance (ESR) spectra and concentration-response curves of direct scavenging activity of remimazolam against multiple free radical Each panel illustrates the following: hydroxyl radical (A), superoxide anion (B), tert-butyl peroxyl radical (C), tert-butoxyl radical (D), ascorbyl free radical (E), singlet oxygen (F), nitric oxide (G), DPPH (H), and tyrosyl radical (I). Remimazolam scavenged hydroxyl radicals (A), superoxide anions (B), ascorbyl free radicals (E), and singlet oxygen (F) in a concentration-dependent manner. Error bars indicate 95% confidence intervals. The vertical axes indicate the ratios of the ESR signal heights (a.u.). Edaravone was used as the positive control for tert-butyl peroxyl (C), tert-butoxyl (D), nitric oxide (G), DPPH (H), and tyrosyl radicals (I).

The ESR spectra of the spin adducts of each radical were examined, and the concentration-response curves are shown in Figure 1. Each spectrum was assigned to the corresponding free radical using the observed hyperfine splitting constant. The IC_50_ values were estimated for each free radical (Table 2). Due to its poor water solubility, it was impossible to conduct an ESR study of midazolam against ascorbyl free radicals, singlet oxygen, nitric oxide, and tyrosyl radicals.

**Table 2:** 50% inhibitory concentration and reaction rate constants remimazolam and midazolam IC_50_ - 50% inhibitory concentration; *k*_G-CYPMPO_ - reaction rate constant of G-CYPMPO with superoxide anion; *k*_EDV_ - reaction rate constant of edaravone with ascorbyl free radical; *k*_OH-TEMP_ - reaction rate constant of OH-TEMP with singlet oxygen –signified no scavenging activity observed; † signified that midazolam was not evaluated because of its poor water solubility under experimental pH conditions

Free radical species	IC_50_ (mM)		Reaction rate constant (M^-1^s^-1^)	
	remimazolam	midazolam	remimazolam	midazolam
Hydroxyl radical	4.2× 10^-2^	1.4 × 10	4.2 × 10^10^	1.3× 10^8^
Superoxide anion	5.8 × 10^-1^	–	8.6 k_G-YPMPO_	–
tert-Butyl peroxyl radical	–	–	–	–
tert-Butoxyl radical	–	8.4	–	0.46 k_G-CYPMPO_
Ascorbyl free radical	5.4 × 10^-1^	†	3.1 × 10^-1^ k_EDV_	†
Singlet oxygen^†^	8.8 × 10^-2^	†	1.9 × 10 k_OH-TEMP_	†
Nitric oxide	–	†	–	†
DPPH	–	–	–	–
Tyrosyl radical	–	†	–	†

Hydroxyl radical 

Remimazolam scavenged hydroxyl radicals in a dose-dependent manner, with an IC_50_ of 0.042 (standard error=0.015) mM (p=0.006; Figure 1A). Because the *k*_*G-*CYPMPO_ for hydroxyl radicals is already known, *k*_RMZ_ was estimated to be 4.2 × 10^10^ (1.5 × 10^10^) M^-1^s^-1^. Midazolam also scavenged hydroxyl radicals in a concentration-dependent manner, with an IC_50_ of 14 (7) mM (p=0.049) and a *k*_MDZ_ of 1.3 × 10^8^ (0.6 × 10^8^) M^-1^s^-1^.

Superoxide anion 

Remimazolam scavenged the superoxide anions in a concentration-dependent manner (Figure 1B). IC_50_ was 0.58 (0.37) mM (p=0.037) with *k*_RMZ_ /*k_G_*_-YPMPO_ = 8.6 (5.5). Midazolam did not scavenge superoxide anions.

Tert-butyl oeroxyl radical 

Neither remimazolam (Figure 1C) nor midazolam scavenged tert-butyl peroxyl radicals.

Tert-butoxyl radical 

Remimazolam scavenged tert-butoxyl radical only at high concentrations (Figure 1D). However, the reaction rate constant was not estimated. Midazolam scavenged tert-butoxyl radicals in a dose-dependent manner with an IC_50_ of 8.4 (2.5) mM (p=0.003) with *k*_MDZ_/*k_G_*_-CYPMPO_=0.46 (0.14).

Ascorbyl free radical 

Remimazolam directly scavenged ascorbyl free radicals in a concentration-dependent manner (IC_50_=0.54 (0.22) mM) (Figure 1E; p=0.009). The kRMZ was estimated to be 0.31 (0.13) *k*_edaravone_. 

Singlet oxygen 

Remimazolam scavenged singlet oxygen in a concentration-dependent manner, with an IC_50_ of 0.088 (0.013) mM (Figure 1F; p<0.001). *k*_RML_/*k*_4-OH_TEMP_ was estimated to be 19 (3).

Nitric oxide 

Remimazolam did not scavenge nitric oxide (Figure 1G).

DPPH 

Neither remimazolam nor midazolam scavenged DPPH (Figure 1H).

Tyrosyl radical 

Remimazolam did not scavenge the tyrosyl radical (Figure 1I).

## Discussion

**Figure 2:**
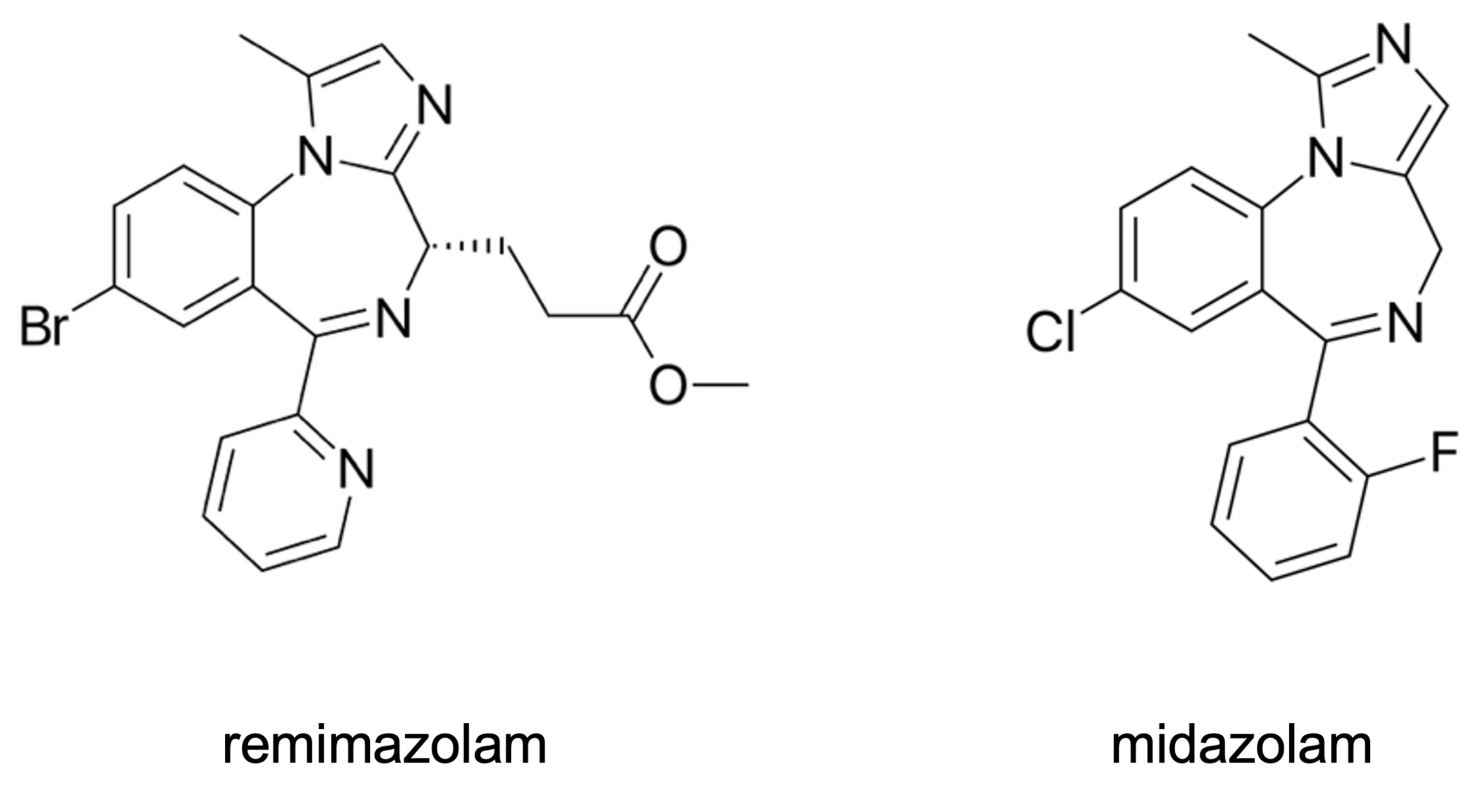
Chemical structural formulae of remimazolam (left) and midazolam (right)

Remimazolam (C_21_H_19_BrN_4_O_2_, 439.31 g/mol; CAS Registry Number® 308242-62-8; Figure 2 left) has a chemical structure similar to midazolam (C_18_H_13_ClFN_3_, 325.77 g/mol; 59467-70-8; Figure 2 right). However, it possesses an ester-linked side chain attached to the diazepine ring, making it an ultrashort-acting intravenous drug that is rapidly converted to an inactive metabolite primarily by liver tissue esterases [2]. Therefore, due to its pharmaceutical characteristics, such as a lower likelihood of prolonged hypnotic effects after discontinuation and more rapid reversal by the antagonist flumazenil compared to midazolam, remimazolam is used as a safer intravenous anesthetic, particularly for older patients and those with unstable hemodynamics.

This study illustrates the concentration-dependent direct radical scavenging activity of remimazolam against multiple free radicals, including hydroxyl radicals, superoxide anions, and nitric oxide. Midazolam also scavenges the hydroxyl radicals and tert-butoxyl radical in a concentration-dependent manner. Additionally, remimazolam exhibited higher free radical scavenging activity than midazolam against hydroxyl radical and superoxide anion. Furthermore, its reaction rate constant for the hydroxyl radical that induced the most severe damage in living organisms was comparable to that of edaravone, the only clinically available free radical scavenger (5.2 × 10^10^ M^-1^s^-1^) [16]. Midazolam exhibits lower antioxidant activity than propofol and dexmedetomidine [23].

Antioxidant effects of various drugs have been reported. However, in many cases, what is evaluated as oxidative stress is oxidized byproducts by reactive oxygen species; e.g., lipid oxidation products such as malondialdehyde, 4-hydroxynonenal, and F2-isoprostane, or DNA oxidation products, e.g., 8-hydroxy-2'-deoxyguanosine. Additionally, antioxidant enzymes such as GSH-Px, catalase, and SOD (as endogenous antioxidants); reducing or oxidizing thiols such as glutathione and cysteine; and antioxidants such as water- or fat-soluble vitamins were measured. Electrochemical measurements, such as oxidation-reduction potential, derivatives of reactive oxygen metabolites, and biological antioxidant potential, were also performed [24]. Although antioxidant activity was examined based on these results, it remains unclear whether each drug exhibits direct scavenging activity against specific types of free radicals or not.

ESR is the only method that can directly identify and quantify free radicals. We established a unique experimental system that utilizes ESR to evaluate the direct free radical scavenging activity of various drugs [16-19]. Generally, most intravenous anesthetics possess antioxidant properties; however, volatile anesthetics have been reported to temporarily increase oxidative stress during prolonged surgical procedures [24, 25]. Using ESR, the potent free radical scavenging activity of propofol was reported; propofol strongly scavenges hydroxyl radicals but not superoxide anions [26, 27]. Similarly, the radical-scavenging activities of midazolam [28] and remimazolam [29] have been reported using ESR. However, they only examined scavenging activities against hydroxyl radical and superoxide anion. They did not report the reaction rate constants for each free radical species.

In this study, remimazolam was found to possess a higher free radical scavenging activity against hydroxyl radicals and superoxide anions than midazolam. This novel finding is expected to be crucial in the future for elucidating the mechanisms underlying the differences in the incidence of POD between remimazolam and midazolam. Furthermore, for patients with preoperative risk factors for POD and those undergoing highly invasive surgery that induces excessive oxidative stress [30], remimazolam may be a better option because it can reduce POD by directly scavenging free radicals or suppressing oxidative stress caused by perioperative ischemia-reperfusion injury. A randomized controlled trial is required to evaluate the effect of remimazolam on the incidence of POD.

The present study has some limitations. First, the possible influence of intra- and extracellular electrolytes, enzymes, and other metabolites on the scavenging activity was not considered. Second, we were able to obtain the absolute reaction rate constants for only hydroxyl radicals. Third, experiments involving the in vivo generation of free radicals were not conducted.

## Conclusions

Remimazolam exhibits concentration-dependent scavenging activity against multiple free radicals. Notably, it demonstrates stronger scavenging activity against hydroxyl and superoxide anions. This contributes to its overall antioxidant capacity and superiority in clinical use over midazolam. It has the potential to be a key drug in a novel antioxidant strategy in anesthesia to prevent POD.
